# Zero value-added tax on fruits and vegetables: beyond health and fiscal standards

**DOI:** 10.1017/S1368980024001617

**Published:** 2024-09-30

**Authors:** Niñoval F Pacaol

**Affiliations:** College of Education – Secondary Laboratory School, Eastern Visayas State University, Tacloban City, Leyte, Philippines

In a recent journal commentary, the authors explored the institutional friction surrounding the Dutch government’s proposal to reduce the value-added tax (VAT) on fruits and vegetables to zero percent, highlighting the differing perspectives of fiscal and health experts^(1)^. The article succinctly discussed the food classifications that may inform the definitions for designing VAT measures on fruits and vegetables and exploring more compelling (or unhealthy) food taxation schemes. Both government declarations and scholarly publications are necessary to bring this issue to the public and research community. However, the current effort is restrictive and selective, focusing on personal and collective well-being without considering climate change mitigation and animal welfare. The approach of fiscal and health experts, based on the commentary, fails to account for the whole food system. Public health nutrition policy should therefore go beyond the economic-health policy dichotomy.

Fundamentally, a zero-VAT policy on fruits and vegetables will make plant-based food items more inclusive and accessible to low-income families and, by extension, address the ‘Hunger-Obesity Paradox’ in Dutch and European societies, especially in developing countries like the Philippines, leading to a productive and healthy working population. Nonetheless, the merit and cost of the intended legislation are not unidirectional and limited to socio-economics; instead, they carry multi-consequential outcomes. When public health nutrition is placed at the centre of the issue, it recognises the potential for sustainable and long-term returns amidst the challenges of intercontinental health hazards due to climate change^(2)^. The Dutch Congress and countries with the same premeditated legislative agenda shall treat it not only as economic regression and a mere step though monumental to address the lived conditions of the poor, who lack the economic means to significantly purchase fruits and vegetables – a struggle uncommon to middle- and upper-income families, but also an integrated support for sustainable development amidst the planetary scale of changing climate.

The move for zero VAT is cost-effective and responsive to planetary health, as people would have a variety of food choices other than meat, whose production and consumption contribute to greenhouse gas emissions and global warming. The role of food intake in climate change is often ignored or undiscussed in lawmaking proceedings, except for the economic and health benefits that may follow from such legislation. If people dramatically increase their consumption of fruits and vegetables and adopt a plant-rich diet, it could address the potential threat of diseases from factory-farmed animals, which could cause a large-scale health crisis. This argument is not a slippery slope but rather involves risk assessment and forecasting, reflecting the expert opinion of scholars in the post-Covid-19 pandemic. Both the Dutch government and consultative experts (i.e. fiscal and health) must adopt an interdisciplinary approach for a holistic and comprehensive analysis, even beyond the language of law, economics or health, as long as data and empirical evidence are considered, such as the link between fruits and vegetables and climate change – a relationship that has a direct impact on our public health and nutrition. By adopting a zero VAT policy on fruits and vegetables, we can take significant steps towards achieving a healthier, more equitable and sustainable future.

## References

[1] Hagenaars LL , Fazzino TL & Mackenbach JD (2024) Giving fruits and vegetables a tax break: lessons from a Dutch attempt. Public Health Nutr 27, e70. 38356382 10.1017/S1368980024000442 PMC10966848

[2] Emergency Nutrition Network (2022) Nutrition and Climate Change - Current State of Play: Scoping Review. Kidlington, Oxfordshire: Emergency Nutrition Network.

